# Phylogenetically Distant *BABY BOOM* Genes From *Setaria italica* Induce Parthenogenesis in Rice

**DOI:** 10.3389/fpls.2022.863908

**Published:** 2022-07-14

**Authors:** Lovepreet Singh Chahal, Joann A. Conner, Peggy Ozias-Akins

**Affiliations:** ^1^Institute of Plant Breeding, Genetics, and Genomics, University of Georgia, Tifton, GA, United States; ^2^Department of Horticulture, University of Georgia, Tifton, GA, United States

**Keywords:** apomixis, parthenogenesis, *BABY BOOM (BBM)*, rice, *Setaria italica*, AP2 transcription factor

## Abstract

The combination of apomixis and hybrid production is hailed as the holy grail of agriculture for the ability of apomixis to fix heterosis of F_1_ hybrids in succeeding generations, thereby eliminating the need for repeated crosses to produce F_1_ hybrids. Apomixis, asexual reproduction through seed, achieves this feat by circumventing two processes that are fundamental to sexual reproduction (meiosis and fertilization) and replacing them with apomeiosis and parthenogenesis, resulting in seeds that are clonal to the maternal parent. Parthenogenesis, embryo development without fertilization, has been genetically engineered in rice, maize, and pearl millet using *PsASGR-BABY BOOM-like* (*PsASGR-BBML*) transgenes and in rice using the *OsBABY BOOM1* (*OsBBM1*) cDNA sequence when expressed under the control of egg cell-specific promoters. A phylogenetic analysis revealed that *BABY BOOM (BBM)/BBML* genes from monocots cluster within three different clades. The *BBM*/*BBML* genes shown to induce parthenogenesis cluster within clade 1 (the ASGR-BBML clade) along with orthologs from other monocot species, such as *Setaria italica*. For this study, we tested the parthenogenetic potential of three *BBM* transgenes from *S. italica*, each a member of a different phylogenetic BBM clade. All transgenes were genomic constructs under the control of the *At*DD45 egg cell-specific promoter. All *SiBBM* transgenes induced various levels of parthenogenetic embryo development, resulting in viable haploid T_1_ seedlings. Poor seed set and lower haploid seed production were characteristics of multiple transgenic lines. The results presented in this study illustrate that further functional characterization of *BBM*s in zygote/embryo development is warranted.

## Introduction

Two processes, meiosis and fertilization, are fundamental to sexual reproduction, the primary mode of reproduction in flowering plants. Meiosis, through the processes of mega-sporogenesis and micro-sporogenesis, leads to the formation of four megaspores from a single megaspore mother cell and four microspores from a single pollen mother cell. During meiosis, recombination between homologous chromosomes leads to the creation of new genetic combinations in the resulting gametes, manifesting in the progeny in the form of new genetic variation (Mercier et al., 2015). In the ovule, one megaspore, usually located at the chalazal end, undergoes mega-gametogenesis which encompasses three mitotic divisions and leads to the formation of a seven-celled, eight nucleate female gametophyte (embryo sac). The remaining three megaspores degenerate (Drews and Koltunow, 2011). Micro-gametogenesis, which involves two mitotic divisions, leads to the formation of the male gametophytes (pollen) from all four microspores (Borg et al., 2009). The egg cell and sperm cell, having reduced number of chromosomes (n) and present in the embryo sac and pollen, respectively, fuse during the process of fertilization to restore the chromosome number/ploidy of the zygote to 2n. The zygote develops into an embryo, which acts as a carrier of genetic information to the next generation.

Apomixis, asexual reproduction through seed, is an exception to sexual reproduction and results in progeny that are clonal to the maternal parent (Asker and Jerling, 1992). Apomixis achieves maternal clonality by bypassing the processes of meiosis and fertilization. The apomictic process is further defined by multiple pathways. In adventitious embryony, a type of sporophytic apomixis, somatic cell(s) in the ovule assumes an embryonic fate and directly develops into embryo(s) through mitotic-like divisions without going through an intermediate phase of embryo sac formation. In contrast, during gametophytic apomixis, female gametophytes develop; however, the development does not involve meiosis but rather the process of apomeiosis. To maintain ploidy in succeeding generations, the apomictic female gamete directly develops into an embryo through the process of parthenogenesis.

Apospory, a subtype of gametophytic apomixis, is the prevalent form of apomixis in the natural apomict *Cenchrus squamulatus* (syn. *Pennisetum squamulatum*) and *C. ciliaris*, close relatives of cultivated and sexually reproducing crop species *C. americanus* (syn. *P. glaucum)*. In apospory, the nucellar cell(s) adjacent to the megaspore mother cell (MMC) in the ovule becomes aposporous initials, undergoes 2 rounds of mitosis, and develops as an aposporous embryo sac containing an egg cell with unreduced chromosome number (2n), synergid(s), and polar nuclei. The embryo develops without fertilization through the process of parthenogenesis. A single, large, hemizygous region called the apospory specific genomic region (ASGR), approximately 50 Mb in size (Akiyama et al., 2004), is responsible for apospory in *C. squamulatus* as evident from the co-segregation of ASGR-linked markers with the apospory phenotype (Ozias-Akins et al., 1998). The ASGR is highly conserved and syntenic between *C. squamulatus* and *C. ciliaris* (Roche et al., 1999; Goel et al., 2006). Apomeiosis and parthenogenesis can be unlinked through rare recombination of the ASGR in *C. ciliaris* (Conner et al., 2013). Sequencing of ASGR-linked BAC clones identified multiple *ASGR-BABY BOOM-like* (*ASGR-BBML*) genes in both species (Roche et al., 2002; Conner et al., 2008).

The *ASGR-BBML*s belong within the *BABY BOOM* (*BBM*) clade, within the euANT lineage of AINTEGUMENTA (ANT), within the larger AP2 family of transcription factors. Members of the euANT group control important roles in establishing and maintaining the activity of diverse meristems within the plant, with *BBM*s playing a role in embryogenesis (Horstman et al., 2014). *BBM*s, like other AP2 subfamily members, contain two AP2 DNA binding domains along with a bbm-1 motif characteristic to BBM/BBML proteins (El Ouakfaoui et al., 2010). Studies have provided evidence for the role of BBM/BBML as pluripotency factors, playing key roles in somatic, as well as sexual embryogenesis. *BBM*s from eudicot sexually reproducing species *Arabidopsis thaliana BBM (At5G17430)*, *Brassica napus BBM1 (AAM33800.1)*, and *B. napus BBM2 (AAM33801.1)* express in zygotic and *in vitro*-induced microspore culture embryos from early globular to later stages of seed development. *p35S:AtBBM* and *pUBI:BnBBM1/2* constructs induce somatic embryogenesis (SE) on shoot apex, leaf and cotyledon blade margins, and petioles in both *Arabidopsis* and *B. napus* (Boutilier et al., 2002). Ectopic expression of *BBM* transgenes improved transformation and regeneration efficiencies in *Theobroma cacao* (Florez et al., 2015), sweet pepper (Heidmann et al., 2011), *Populus tomentosa* (Deng et al., 2009), and *Zea mays* (Du et al., 2019). The combination of a *ZmBBM* and *Wuschel* (*Wus*) cassette greatly improved transformation efficiencies for *Oryza sativa*, *Sorghum bicolor*, *Saccharum officinarum*, and *Z. mays* (Lowe et al., 2016, 2018).

Apospory specific genomic region-*BABY BOOM-like* (*ASGR-BBML*) expresses in the egg cell of mature embryo sacs prior to anthesis in *C. ciliaris* (Ke et al., 2021). Similarly, sexually reproducing pearl millet carrying a *PsASGR-BBMLpromoter:GUS* cassette displayed GUS signal in egg cells one day prior to anthesis and up to three days after anthesis in the developing embryo (Conner et al., 2015). The transfer of *PsASGR-BBML* cDNA and genomic DNA cassettes under the control of either the native promoter or an egg cell-specific *At*DD45 (*At*2g21740) promoter (Steffen et al., 2007) from *Arabidopsis* induced parthenogenesis in pearl millet, rice, maize, and tobacco at various levels (Conner et al., 2015, 2017; Zhang et al., 2020). Similarly in rice, *OsBBM1* (LOC_Os11g19060), closely related to *PsASGR-BBML*, triggered somatic embryogenesis on seedling leaves when driven by an ubiquitin promoter and induced parthenogenesis when expressed using the egg cell-specific *At*DD45 promoter (Khanday et al., 2019).

The role of *BBM/BBML* genes in parthenogenesis in sexual plants and somatic embryogenesis relies on changing the expression pattern, either by overexpression or developmental timing. Multiple *BABY BOOM* (*BBM*) genes have been identified in sexually reproducing monocot plant species for which genomic resources have been developed, such as *O. sativa* (Khanday et al., 2019), *Z. mays* (Lowe et al., 2016; Du et al., 2019), *Setaria italica*, *Brachypodium distachyon*, *Triticum aestivum* (Bilichak et al., 2018), and *S. bicolor* (Yavuz and Çalışkan, 2021), indicating a possible conserved function for *BBM/BBML* genes. However, monocot *BBM/BBML* cluster into distinct clades with the parthenogenetic potential of *BBM/BBML* genes having only been confirmed for genes within the ASGR-BBML clade (Conner et al., 2015; Khanday et al., 2019). *S. italica* (foxtail millet) is a genome-sequenced monocot species which retains a large degree of collinearity with the rice genome and is also a close diploid relative of *C. ciliaris*, switchgrass, and pearl millet. *S. italica* also contains a unique *BBM* gene copy per phylogenetic BBM/BBML clade. This study demonstrates that all three Si*BBM* genes, regardless of phylogenetic BBM/BBML clade, can induce parthenogenetic embryo development in the monocot model crop rice when expressed from an egg cell-specific promoter. These results greatly expand the potential number of *BBM/BBML* genes that can be engineered for use in creating haploid seedlings and engineering apomixis in sexual plants.

## Materials and Methods

### Sequence Extraction, Sequence Alignment, and Phylogenetic Analysis

The OsBBM1(LOC_OS11g19060 - O.sativa v7.0) protein sequence was used to extract similar protein sequences from Phytozome v12 (Goodstein et al., 2012). Proteins having sequence similarity greater than 34% were extracted for *Z. mays*, *S. italica*, *S. viridis*, *Panicum halli*, *P. virgatum*, *O. sativa*, *B. distachyon*, *S. bicolor*, *Glycine max*, *Medicago truncatula*, and *Arabidopsis thaliana*. The proteins were downloaded both from Phytozome and NCBI. *Gossypium raimondii*. *C. ciliaris*, *C. squamulatus*, and *B. napus* BBM protein sequences were extracted from NCBI Genbank. Protein sequences were aligned using ClustalW (default settings) in MEGA7 (Kumar et al., 2016). The aligned sequences were inspected for the conserved bbm-1 motif (consensus sequence of GLSMIKNWLR) prior to the first AP2 domain (El Ouakfaoui et al., 2010). Forty-two BBM/BBML sequences, containing the bbm-1 motif, were aligned using ClustalW (default settings) in MEGAX (Kumar et al., 2018) and a phylogenetic tree was constructed using the Maximum Likelihood method and the JTT matrix-based model (Jones et al., 1992) with 500 bootstrap replications.

### Transformation Constructs and Plant Growth

Three genomic *SiBBM* cassettes were placed in a pCambia1300-*DD45* promoter base vector with detailed descriptions of the Arabidopsis *DD45* cloning described in Conner et al., 2017. The pCambia1300-*DD45* base vector contains a unique *Afl*II site inserted prior to the start codon for cloning purposes. The Arabidopsis *DD45* promoter drives gene expression in egg cells of Arabidopsis and rice (Albertsen et al., 2013; Lawit et al., 2013; Ohnishi et al., 2014). All *BBM* cassettes were PCR amplified from *S. italica* cv. Yuga1 using primers described in Supplementary Table 1: “purpose – construct” and generated using the Geneious Prime software (Biomatters Ltd., Auckland, New Zealand). The PCR reaction consisted of 25 ng of *S. italica* genomic DNA, 1X PrimeSTAR GXL Buffer, 200 μM dNTP, 0.2 μM primers, and 0.625U PrimeSTAR GXL DNA polymerase in a 25-μl reaction (Takara Bio United States, Inc., Mountain View, CA, United States). The annealing temperature for each reaction was based on the primer with the lowest Tm and 35 cycles of amplification. Each forward primer used for the *SiBBM* cassettes contained an *Afl*II site prior to the start codon. The *gSiBBM1* reverse primer was located 515 bp after the stop codon. Both *gSiBBM2* and *gSiBBM3* contained an internal *Afl*II site and a 2-step cloning protocol was needed. Two sets of primers were created (5pF/R and 3pF/R) for each gene which overlapped the internal *Afl*II site. The gSiBBM2-3pR primer is located 622 bp from the stop codon and contains a *Bam*HI restriction site. The gSiBBM3-3pR primer is located 663 bp from the stop codon and contains a *Sal*I restriction site. The resulting construct amplicons were cloned into the pCR-Blunt vector (Invitrogen, Carlsbad, CA, United States) according to the manufacturer’s instructions. Plasmids with inserts were extracted using the QIAprep Spin Miniprep Kit (QIAGEN Inc., Germantown, MD, United States) and verified by sequencing (Psomagen, Rockville, MD, United States). The native *SiBBM* 3′ UTRs were based on transcriptomic databases at Phytozome^1^ and NCBI^2^. pCambia1300-*DD45* and gSiBBM1 TOPO plasmids were digested with *AlfII/Sal*I and *AlfII/Xho*I, respectively, and the desired fragments were ligated, transformed, and colony analyzed. A two-step cloning method was used for the gSiBBM2 and gSiBBM3 constructs. pCambia1300-*DD45* and a gSiBBM2-3pF/R TOPO plasmids were digested with *AlfII/Bam*HI while pCambia1300-*DD45* and a gSiBBM3-3pF/R TOPO plasmids were digested with *AlfII/Sal*I. The desired fragments were ligated, transformed, and colony analyzed. The second cloning step was to digest pCambia1300-*DD45*:gSiBBM2-3pF/R and pCambia1300-*AtDD45*:gSiBBM3-3pF/R, gSiBBM2-5pF/R TOPO, and SiBBM3-5pF/R TOPO plasmids with *Afl*II. The desired fragments were ligated, transformed, and colony analyzed for the insert. The constructs - pCambia1300-*DD45-gSiBBM1* (based on Seita.8G107100: denoted as *gSiBBM1*), *pCambia1300-DD45-gSiBBM2* (based on Seita.5G415800: denoted as *gSiBBM2*), and pCambia1300-*DD45-gSiBBM3* (based on Seita.1G232200: denoted as *gSiBBM3*) - were verified by sequencing (Psomagen) and sent to the Plant Transformation Facility, Cornell University, Ithaca, NY, United States, or the Plant Transformation Facility, Iowa State University, Ames, IA, United States, for *Agrobacterium tumefaciens*-mediated transformation of rice (*Oryza sativa* Nipponbare) callus and subsequent plantlet regeneration.

Independent lines of plantlets were received and shifted to soil (a mixture of 1:1 volume of ProMix BX-M soil:OIL-DRI clay absorbent supplemented with 3.0 g of Everris Nursery Mix 18-5-12 fertilizer and 1.75 g of Encap Fast Acting Iron per 4-in square pot), hardened, and matured in the NESPAL greenhouse facility, Tifton, GA maintained at 24–29°C with natural lighting. Plants were bottom watered with low pH water (pH 5.2–5.8) and additionally fertilized with “Miracle-Gro^®^ Plant Food” and “Ferti-Lome Chelated Liquid Iron and Other Micro Nutrients” as needed.

### Plant DNA Extraction and Transgene Evaluation

The DNA was extracted from transgenic lines using a CTAB method (Conner et al., 2013) and evaluated by PCR in the T_0_ plants for the full length Si*BBM* coding regions and the hygromycin selectable marker in the T_1_ plants (Supplementary Table 1: “purpose – genotyping”). The PCR reactions consisted of 2 μl DNA, 1X PrimeSTAR GXL Buffer, 200 μM dNTP, 0.2 μM primers, 0.625U PrimeSTAR GXL DNA polymerase in a 25-μl reaction (Takara Bio United States, Inc., Mountain View, CA, United States). The annealing temperature was based on the lowest Tm for the primers used and a 35-cycle amplification. The results were visualized on a 1% agarose gel stained with EtBr or GelRed (Sigma-Aldrich, Inc., St. Louis, MO, United States).

### Rice Ovary, Young Leaf Tissue, and Bud Collection for RNA Extraction

T_0_ rice ovaries were collected approximately one day prior to anthesis. Spikelets were removed from the panicle and placed in a Petri dish for dissection. The palea, lemma, glume, and lodicule were removed using a scalpel and tweezers, leaving behind the carpel and stamen. Stigmas and anthers were observed at 10X magnification under a Leica StereoZoom 4 microscope (Leica microsystems, Wetzlar, Germany) and checked for anther dehiscence and pollen grains on the stigmatic tissue. If no anther dehiscence or pollen on stigmas was observed, the stigma was removed and the remaining ovary was collected in RNAlater (Invitrogen Co., Carlsbad, CA, United States) and stored per manufacturer’s instructions or frozen in liquid nitrogen immediately after dissection and stored at −80*^o^*C. Approximately 30 ovaries were collected for a sample.

Both the leaf and the bud tissue were collected from T_0_ plants. Approximately 100 mg of emergent young leaf tissue (less than 6 cm in length) was collected from plants that were at the reproductive phase, but still producing new leaf. To collect an axillary bud tissue, tillers were removed from the plant. The leaf sheaths were removed and the bud tissue was removed with a scalpel. Both the leaf and the bud tissue were collected in 1.5 ml Eppendorf tubes placed in liquid nitrogen and stored at −80*^o^*C.

### RNA Extraction, Reverse Transcription, Non-quantitative RT-PCR, and Transgene Sequence Verification

The RNeasy plant mini kit (QIAGEN Inc., Germantown, MD, United States) was used for RNA extraction. From RNAlater preserved tissues, RNAlater was removed and tissues were disrupted in an RTL buffer supplemented with 2-merceptaethanol by grinding. Tissues stored at −80*^o^*C were ground in liquid nitrogen. One microgram of total RNA was DNase treated (Invitrogen Co., Carlsbad, CA, United States) and used for reverse transcription using the SuperScriptIII first-strand synthesis system (Invitrogen Co., Carlsbad, CA, United States). For non-quantitative RT-PCR reactions (Supplementary Table 1: “purpose - RT-PCR”), 2 μl of first stand cDNA, 1x PrimeSTAR GXL buffer, 200 μM dNTPs, 0.2 μM primer, and 0.625U of PrimerSTAR GXL DNA polymerase were used in a 25-μl reaction (Takara Bio USA, Inc., Mountain View, CA, United States). To verify the sequence and the splicing of the *SiBBM* transgenes, full-length CDS was amplified through RT-PCR. The annealing temperature was based on the lowest Tm for the primers used. The resulting amplicon was cloned into the pCR-Blunt vector (Invitrogen, Carlsbad, CA, United States) according to the manufacturer’s instructions. Plasmids with inserts were extracted using the QIAprep Spin Miniprep Kit (QIAGEN Inc., Germantown, MD, United States) and sequenced (Psomagen).

### Flow Cytometry

A BD Accuri C6 flow cytometer (BD Biosciences, San Jose, CA, United States) was used to measure the ploidy of T_1_ rice leaves and T_1_ embryos within seed with *Paspalum notatum* leaf tissue used as a genome standard (Conner et al., 2017). For leaf flow cytometry, approximately 0.5 cm of the tip of a rice seedling leaf with a similar size of *P. notatum* leaf tissue were chopped together in a small Petri dish with 150 μl of LB01 buffer (15 mM *Tris*, 2 mM Na_2_EDTA, 0.5 mM spermine-4HCl, 80 mM KCl, 20 mM NaCl, 0.1% v/v Triton X-100 pH7.5, and 16 mM ß-mercaptoethanol) with a double-edged razor blade. An additional 150 μl of LB01 was added and the solution passed through a 30-μm CellTrics disposable filter (Partec Munster, Germany). One hundred fifty microliters of RNAse/propidium iodide solution (BD Biosciences) was added to each filtered sample and incubated on ice for at least 15 min prior to analysis.

Mature seed were collected from T_0_ plants for bulk seed flow cytometry (BSFC). The seed were collected randomly from 2 to 4 panicles per plant depending on seed set and placed in water overnight to soften the seed for cutting. The bottom third of each seed, which contains the embryo, was removed and processed similarly to leaf tissue. A pool of up to five cut seed was considered one sample of BSFC. The ploidy level was determined after the samples were analyzed for signal using a BD Accuri C6 flow cytometer and an accompanying software. Background signal was removed through gating of signals that only exhibited strong correlation between FL2-A and FSC-A plotted on a logarithmic scale. The ploidy level was determined by plots generated on a linear Y-axis count number and a logarithmic X-axis FL2-A signal. Diploid wild-type rice and the *P. notatum* standard were run individually to give base peaks. The haploid rice signals show a peak FL2-A signal which is half of the diploid peak.

### Parameters Calculated for T_1_ Generation

T_1_ seed set was calculated as percentage of florets that developed mature seed on two or more randomly selected primary or secondary panicles over the total number of florets on the panicles. The germination rate was calculated as the total number of germinated seed with root and shoot growth over the number of seed placed on media. The number of diploid and haploid seedlings was determined by the individual flow cytometry of T_1_ seedling leaf tissue.

### Ovary Fixation and Clearing

For fixation, the rice carpels were collected similar to RNA ovary collection and placed into FAA (3.7% formaldehyde, 5% acetic acid, 47.5% ethanol) for 2 days. Carpels were dehydrated in an ethanol series of 70, 85, and 100% ethanol, each for 2 h, followed by overnight incubation in fresh 100% ethanol. For clearing, a methyl-salicylate (MS) series was carried out which comprised keeping ovaries in 2:1 ethanol:MS, 1:1 ethanol:MS, 1:2 ethanol:MS, and then in 100% MS, each for 2 h. The ovaries remained in 100% MS for at least 2 days before mounting, observation, and tabulation using a Zeiss Axioskop 2 plus microscope (Zeiss, Thornwood, NY, United States) with a differential interference contrast (DIC) microscopy.

Confocal images of MS cleared ovaries were obtained using a Nikon C2 + Ti2-E inverted confocal system (Nikon Instruments INC., Melville, NY, United States). Images were captured with auto florescence generated from merging DAPI (laser/excitation 405 nm:emission 425–475 nm) and FITC/GFP (laser/excitation 488 nm:emission 489–720) signals with image editing using the NIS-Elements software (Nikon Instruments INC., Melville, NY, United States).

## Results

### Phylogenetic Analysis of *BABY BOOM (BBM)/BABY BOOM-Like (BBML)* Genes

**FIGURE 1:**
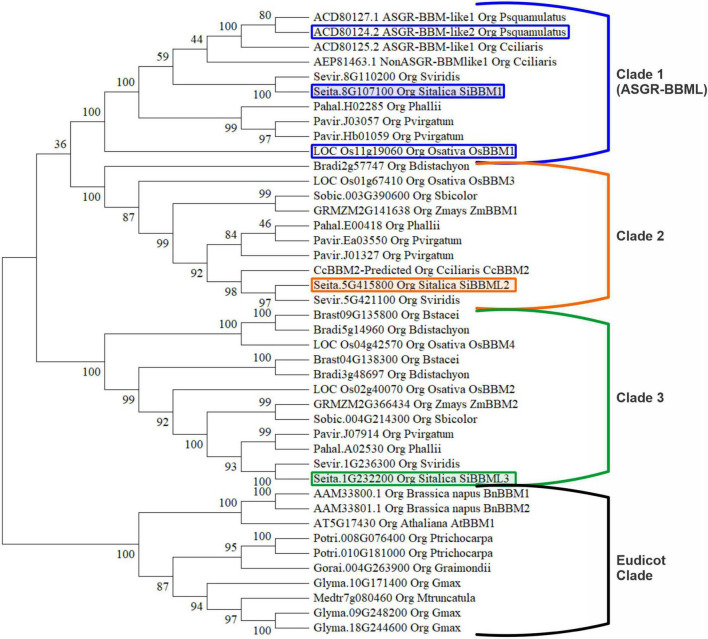
Phylogenetic analysis of 42 *BABY BOOM* (BBM)/*BABY BOOM-like* (BBML) proteins. The phylogenetic tree displays four distinct clades, including a eudicot clade and three monocot clades. The encircled and colored proteins are the *Setaria italica* BBM used in this study. The encircled proteins in clade 1 denotes BBM/BBML proteins which already had been shown to induce parthenogenesis.

Forty-two BBM/BBML protein sequences, thirty-two monocot and ten eudicot, were included for phylogenetic analysis. The analysis included BBM/BBML sequences from sexually reproducing monocot species, *S. bicolor, Z. mays*, *B. distachyon, B. stacei*, *S. italica*, *S. viridis*, *P. hallii*, *P. virgatum*, and *O. sativa*, from apomictic species *C. squamulatus* and *C. ciliaris*, and eudicot sexual species *A. thaliana*, *G. raimondii*, *M. truncatula*, *B. napus*, *P. trichocarpa*, and *G. max*. The phylogenetic tree, inferred by the maximum likelihood method based on a ClustalW alignment in MEGAX, displayed four distinct clades. There were a total of 971 positions in the final dataset and the tree with the highest log likelihood is displayed in Figure 1. The phylogenetic analysis displays a Eudicot clade of BBM/BBML proteins and three distinctive clades of monocot BBM/BBML proteins labeled one through three. Clade 1 (ASGR-BBML clade) contains the apomictic derived ASGR-BBML proteins along with rice LOC_Os11g19060 (OsBBM1). Both of these genes have been shown to promote parthenogenesis in rice when expressed with the Arabidopsis DD45 promoter (Conner et al., 2017; Khanday et al., 2019). The three BBM/BBML proteins were identified from the *S. italica* genome, each belong to a separate monocot clade, and were chosen for a transgene analysis.

### Transgenic T_0_ Line Evaluation

**FIGURE 2:**
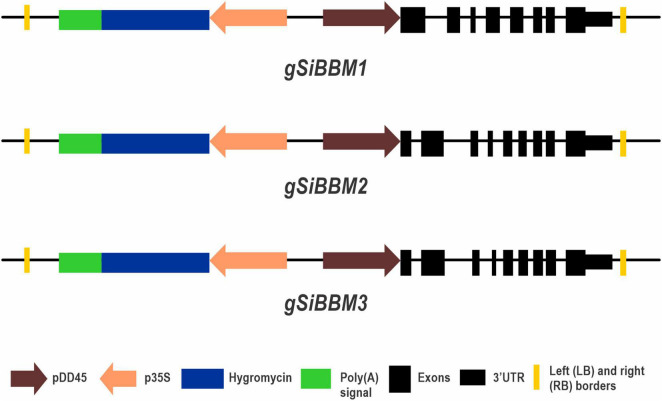
*gSiBBM* constructs. A diagrammatic figure of the three genomic *SiBBM* constructs used in this study. Transgenic plantlets were selected with hygromycin driven by the CaMV 35S promoter. *SiBBM1* contains 8 exons, while *SiBBM2* and *SiBBM3* have 9 exons. The constructs are not drawn to scale.

**FIGURE 3:**
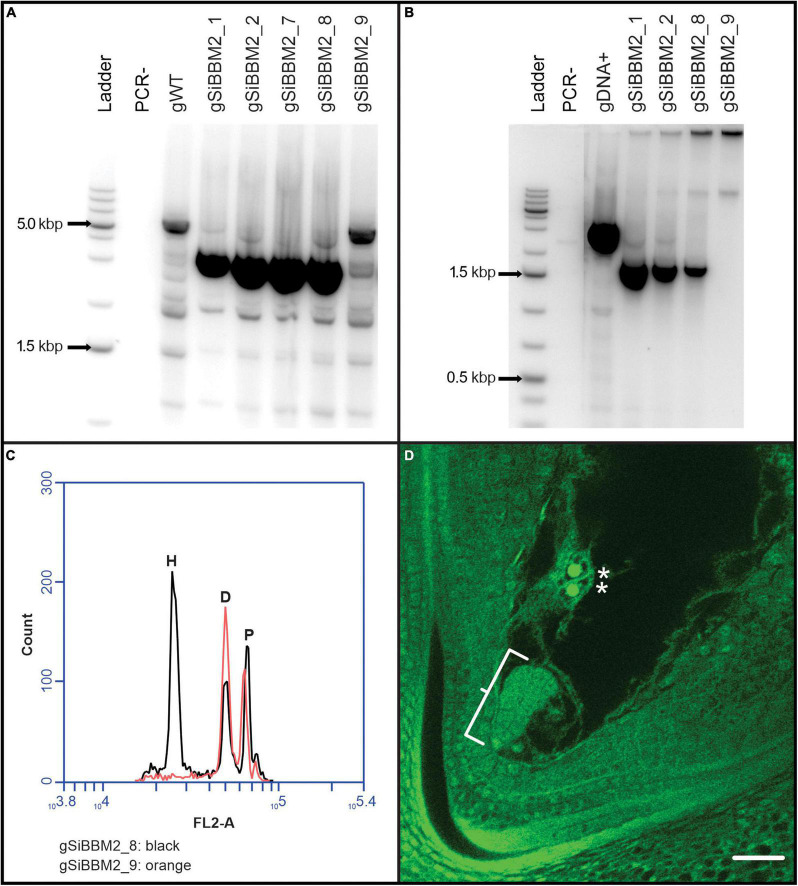
Overview of transgenic T_0_ line evaluation. Analysis of *gSiBBM2* lines **(A–D)**. **(A)** PCR products from transgenic lines, most showing the expected size of the full length g*SiBBM*2 transgene. *gSiBBM2_9*, while positive for the hygromycin plant selectable marker, did not contain a full-length *gSiBBM2* transgene. The PCR- lane is a negative control with no DNA added to the PCR reaction tube while the gWT lane is DNA isolated from an untransformed wild-type *Oryza sativa* Nipponbare plant. **(B)** Non-quantitative RT-PCR expression of the *gSiBBM2* transgene in ovary tissue collected prior to fertilization. **(C)** Bulk seed flow cytometry results as an overlay from two samples of *gSiBBM2* lines, one with (black) and without (red) the *gSiBBM2* transgene. The H peak corresponds to rice embryo haploid (N) signal, the D peak corresponds to the rice embryo diploid (2C) signal, and the P peak corresponds to the genome standard *P. notum* diploid (2C) signal. **(D)** Confocal image of a parthenogenic embryo in rice induced by the *gSiBBM2* transgene. The parthenogenetic embryo is shown in the bracket, while the presence of unfused polar nuclei (starred) denotes that fertilization has not occurred. The scale bar equals 25 μm.

Five, thirty-one, and thirty independent lines were received from the transformation facility and survived to flowering stage for constructs *gSiBBM1*, *gSiBBM2*, and *gSiBBM3*, respectively (Figure 2). The cumulative data for the analysis of the three *gSiBBM* lines are shown in Supplementary Table 2. All lines were genotyped for a full-length transgene, yielding five positive and no negative lines for *gSiBBM1* and twenty-four positive each and seven or six negative lines for *gSiBBM2* (*gSiBBM2* lines in Figure 3A) and *gSiBBM3*, respectively. The expression of the transgenes was evaluated by a non-quantitative RT-PCR of RNA from the ovary tissue collected approximately 1 day prior to anthesis. The transgene expression was observed in all g*SiBBM1* lines, and 22 out of 23 *gSiBBM2* and *gSiBBM3* lines assayed (*gSiBBM2* lines in Figure 3B). Full-length transcripts from the ovary tissue for each *SiBBM* construct were cloned and sequenced from a limited number of lines and validated the expected sequence and splicing of the transgenes. The parthenogenetic induction in T_0_ lines was evaluated through bulk seed flow cytometry (BSFC). Depending on seed set, one to four samples containing up to five embryos were bulked into one flow sample and analyzed for haploid signal. A haploid peak associated with a BSFC sample denotes the transgene is inducing parthenogenesis or haploid cell division from sexually reduced embryo sacs. Lines that did not contain a full-length transgene did not display haploid signal (gSiBBM2–9 in Figure 3C). All gSiBBM1 lines, 23 out of 24 gSiBBM2 lines (gSiBBM2–8 in Figure 3C) and 16 out of 19 *gSiBBM3* lines assayed, showed some occurrence of haploid signal with BSFC. While flow cytometry was mostly used to determine parthenogenesis, six gSiBBM2 and eight gSiBBM3 lines were evaluated for parthenogenesis based on visualization of early embryo development prior to fertilization through ovary clearing and microscopy (*gSiBBM2* in Figure 3D).

T_1_ seeds were germinated from T_0_ plant lines for each *gSiBBM* transgene (Table 1). The choice of T_0_ lines included for the T_1_ study included variables, such as total seed available and the amount of haploid signal detected for the T_1_ BSFC analysis, with lines showing increased amounts of haploid signal prioritized. The seed set was calculated for the T_0_ lines chosen for T_1_ germination (Supplementary Table 2). *gSiBBM1* seed set ranged from 28 to 72%, *gSiBBM2* from 39 to 70%, and *gSiBBM3* from 26 to 43%. Wild-type seed set ranged from 85 to 90%. The T_1_ seed were sterilized, germinated on MS plus sucrose media, and evaluated for seedling viability, haploid production, and transgene inheritance. Germination rates ranged from 36 to 94% for *gSiBBM1* lines, from 38 to 72% for *gSiBBM2* lines, and from 68 to 96% for *gSiBBM3* lines. All lines, except for *gSiBBM1*-8B, produced at least 1 viable haploid seedling. The number of seeds containing viable haploid seedlings was variable within each construct, with the best line producing 20, 16, and 30% haploids for *gSiBBM1*, *gSiBBM2*, and *gSiBBM3*, respectively. The seed that did not germinate was also screened with flow cytometry for haploid signal. Both haploid/diploid- and diploid-only signals were identified from non-germinating seed. All lines were able to inherit the transgene both in haploid and diploid offspring (Table 1).

**TABLE 1:** Summary of T_1_ seed/seedling evaluation of *gSiBBM1*, *gSiBBM2*, and *gSiBBM3* lines.

Construct	Line	Seed germinated	Number of viable seedlings	Germination rate (%)	Diploid seedlings	Haploid seedlings	Number of ungerminated seed	Haploid signal in ungerminated seed	Transgene inheritance in diploid offspring
*gSiBBM1*	−6C	50	47	94	37	10	3	Yes	Yes
	−7B	50	42	84	41	1	8	Yes	Yes
	−8B	50	18	36	18	0	32	Yes	Yes
	−4B	50	31	62	28	3	19	Yes	Yes
	−5C	50	38	76	35	3	12	Yes	Yes
*gSiBBM2*	−1	50	31	62	23	8	19	Yes	Yes
	−7	50	19	38	16	3	31	Yes	Yes
	−43	50	36	72	35	2	14	Yes	Yes
	−50	50	31	62	27	4	19	Yes	Yes
	−44	50	35	70	31	4	15	Yes	Yes
	−8	50	32	64	28	4	18	Yes	Yes
*gSiBBM3*	−8	50	45	90	39	6	5	Yes	Yes
	−5	38	34	89	26	8	4	Yes	Yes
	−27	50	48	96	33	15	2	n/a	Yes
	−9	50	34	68	31	3	16	Yes	Yes

The overall plant morphology of T_0_ transgenic lines from *gSiBBM1* and *gSiBBM2* was similar to those of wild-type plants. However, the *gSiBBM3* T_0_ transgenic lines displayed growth irregularities, including a decrease in total height, increased tillers, intermittent awned florets, and a lack of panicle/seed extrusion from the flag leaf. RNA was extracted from young leaf and axillary bud tissue of three *gSiBBM3* transgene positive plants and from one *gSiBBM3* transgene negative plant (*gSiBBM3_2)*. The leaf and bud tissue samples, except for the negative *gSiBBM3_2* line, showed expression of the transgenes (Supplementary Figure 1). An additional 15 seed from T_0_ lines *gSiBBM3_8* and *gSiBBM3_9* and five wild type seed were germinated and grown. After removal of haploid seedlings and ungerminated seed, seven T_1_ *gSiBBM3_8* plants, eight T_1_ *gSiBBM3_9* plants, and 4 wild type plants could be genotyped and phenotyped. Of the seven T_1_ *gSiBBM3_8* plants, two genotyped without the gSiBBM3 transgene had growth patterns similar to the wild-type plants, while five plants with the *gSiBBM3* transgene had the irregular morphology. Of the eight T_1_ *gSiBBM3_9* plants, one genotyped without the *gSiBBM3* transgene had growth patterns similar to the wild-type plants, while the seven plants with the *gSiBBM3* transgene all had irregular morphology.

## Discussion

Studies have determined that the apomictic-locus-derived *PsASGR-BBML* and the sexual-derived, paternally expressed *OsBBM1* can induce parthenogenesis in rice when expressed under the control of the Arabidopsis DD45 egg cell-specific promoter (Conner et al., 2017; Khanday et al., 2019). *PsASGR-BBML* has also been shown to induce parthenogenesis in transgenic sexual pearl millet, maize, and tobacco when expressed with the Arabidopsis DD45 egg cell-specific promoter (Conner et al., 2015, 2017; Zhang et al., 2020). This study was designed to determine if other BBM genes, both similar to and more phylogenetically diverged from *PsASGR-BBML* and *OsBBM1*, could promote parthenogenesis in rice if expressed in egg cells. *S. italica BBM* genes were chosen for this analysis. *S. italica* is a diploid species closely related to *Cenchrus*, has a similar divergence from rice, and contains a single *BBM* gene for each of the three monocot clades identified in a BBM/BBML phylogenetic analysis. Sexual crop species, such as *Z. mays* and *S. bicolor* and the model grass species *B. distachyon*, do not contain a *BBM* gene belonging to clade 1 (PsASGR-BBML), while rice has two *BBM* genes in clade 3.

*SiBBM1* falls within clade 1 (PsASGR-BBML) and shares most similarity to a *Non-ASGR-BBML* gene from *C. ciliaris* (Zeng, 2009). Clade 1 contains BBM/BBML genes from both sexual and apomictic species and has the two *BBM/BBML* genes identified as promoting parthenogenesis in rice. *SiBBM2* falls within clade 2. Clade 2 contains the *C. ciliaris* gene (*CcBBML2*) which was identified as a *de novo* expressed and upregulated gene in the egg cell of apomictic but not sexual *C. ciliaris* prior to pollination (Ke et al., 2021). *CcBBML2* has not yet been mapped to the *C. ciliaris* genome; therefore, its linkage as an apomixis-linked or sexual gene has not been determined. Clade 2 also contains *Z. mays BBM1 (GRMZM2G141638)* and *OsBBM3*. *ZmBBM1* is upregulated during callus induction and *pUBI:ZmBBM1*, in combination with *pNOS:Wuschel* (*Wus*), improves transformation efficiencies in rice, sorghum, sugarcane, and maize (Lowe et al., 2016, 2018). *SiBBM3* falls within clade 3 and includes *ZmBBM2 (GRMZM2G366434)*, recognized as a highly upregulated transcript during callus induction, and demonstrates a 3- to 7-fold increase in transformation efficiency in maize when expressed ectopically (Du et al., 2019).

All *S. italica BBM* (g*SiBBM1*, g*SiBBM2*, and g*SiBBM3*) transgenes induced parthenogenesis in rice. *SiBBM1* becomes the third BBM/BBML member of clade 1 (ASGR-BBML) capable of inducing parthenogenesis in rice. *SiBBM2* and *SiBBM3* become the first BBM members of clades 2 and 3 to be shown to induce parthenogenesis in rice when expressed under an egg cell-specific promoter. This study demonstrates that neo-functionalization of ASGR-BBML or BBM proteins within clade 1, specifically to induce parthenogenesis, did not occur, but rather that expression of *BBM* genes in egg cells prior to fertilization will induce parthenogenesis in rice. Recently, the PARTHENOGENESIS (PAR) allele from apomictic dandelion (*Taraxacum officinale*) was identified. The PAR gene encodes a zinc finger domain protein with an EAR motif and is similar to the ASGR-BBML/BBM genes in that expression of the PAR allele has been modified, in this case by the insertion of a non-autonomous MITE in the promoter region to promote egg cell-specific expression (Underwood et al., 2022). Although the original number of T_0_ lines for selection of T_1_ lines was reduced for the *gSiBBM1* transgene, this construct did not outperform *gSiBBM2* or *gSiBBM3* in percent seed set of the T_0_ plants nor in the ability to produce viable haploid seedlings in the T_1_ generation. While most T_0_ *gSiBBM* lines with a complete transgene produced some level of parthenogenesis, a ‘perfectly penetrant’ line that was highly fertile, produced 50% haploid progeny (from parthenogenesis of maternal egg cells with a single transgene integration) and diploid offspring that were 50–50 transgene positive-negative genotypes (from paternal inheritance from a single transgene integration) was not identified. The rates of T_1_ haploid seedling production ranged from 0 to 20% for *gSiBBM1*, from 4 to 16% for *gSiBBM2*, and from 4 to 32% for *gSiBBM3*. This result is similar to the line variability that was identified when the *PsASGR-BBML* and *OsBBM1* transgenes were used to induce parthenogenesis. The frequency of haploid seedlings from the *OsBBM1* cDNA transgenic lines ranged between 5.8 and 10.5% (Khanday et al., 2019) while rates of 0–16% were identified for the various *PsASGR-BBML* transgenes (Conner et al., 2017). The *SiBBM* lines also shared similar ovary developmental fates as those displayed with rice transgenic lines carrying the various *PsASGR-BBML* transgenes, including ovaries with no expansion, halted ovary expansion prior to milky endosperm development, and callus-like formations (Conner et al., 2017). These similar ovary phenotypes support the conclusion that the BBM proteins within the three monocot clades perform similar functions and alter similar pathways during rice reproduction. Many of the T_1_ *gSiBBM* lines had germination rates below that of wild type, probably due to poor embryo development and similar to what was seen with embryo development in the *PsASGR-BBML* transgenic lines (Conner et al., 2017). A study of cell division of zygotes formed by electro-fusion of rice gametes observed that an increase of *OsBBM1* in zygotes could lead to an inhibitory effect on the progression of zygotic development. These data suggest that excess *OsBBM1* could be harmful to the embryo development and that expression levels of *OsBBML1* in zygotes need to be critically regulated for the optimal zygote development (Rahman et al., 2019).

The phenotypically different vegetative growths identified in the *gSiBBM3* lines lead to the isolation of RNA from leaf and bud tissue, tissue types not associated with the expression pattern of the egg cell-specific *Arabidopsis DD45* promoter. *SiBBM3* transcript was identified in both leaf and bud tissue. Identification of transcripts in leaf tissue from multiple lines containing a *ZsGreen1*-fused *gPsASGR-BBML* cassette in the same pCambia1300-*AtDD45* promoter base vector suggests the *SiBBM* transgenes and all pCambia1300-*AtDD45* promoter base vectors used in the previous studies (Conner et al., 2017; Khanday et al., 2019) may also have ectopic expression of the various *BBM* transgenes which will need to be addressed in further study. The ectopic expression is probably due to the enhanced CaMV 35S promoter located upstream of the *At*DD45 promoter in the pCambia1300 vector backbone which is used to drive expression of the hygromycin resistance plant selectable marker. The 35S promoter-driving selectable marker expression has been previously reported to interfere with the tissue/developmental stage-specific transgene expression (Jagannath et al., 2001; Yoo et al., 2005). Replacement of the CaMV 35S promoter with a plant promoter, such as ubiquitin, may reduce ectopic expression of the *BBM* transgenes. Physical separation of the integration of the plant transformation selection cassette and the *BBM* transgenes through co-transformation, while reducing transformation efficiency, should also reduce the chances of ectopic expression.

This study confirms that all *SiBBM* transgenes can induce parthenogenesis and viable haploid seed production in rice when expressed under an egg cell-specific promoter. Given the conservation of BBM across monocot species, it is likely that any *BBM* transgenic construct will be able to promote parthenogenesis in monocot species when expressed from an egg cell-specific promoter. In rice, the clade origin of the SiBBM did not affect the outcome and thus greatly increases the number of genes with the potential to induce parthenogenesis. Fine tuning the expression level and/or pattern of these transgenes will be critical to increase the penetrance of parthenogenesis, the viable embryo development, and the wild-type level seed set.

## Data Availability Statement

The original contributions presented in this study are included in the article/Supplementary Material, further inquiries can be directed to the corresponding author.

## Author Contributions

PO-A and JC conceptualized this project. Methodology, investigation and analysis was done by LC and JC. JC supervised the project. LC wrote the original draft. All authors revised and approved the manuscript.

## Conflict of Interest

The authors declare that the research was conducted in the absence of any commercial or financial relationships that could be construed as a potential conflict of interest.

## Publisher’s Note

All claims expressed in this article are solely those of the authors and do not necessarily represent those of their affiliated organizations, or those of the publisher, the editors and the reviewers. Any product that may be evaluated in this article, or claim that may be made by its manufacturer, is not guaranteed or endorsed by the publisher.

## Footnotes

1
https://phytozome-next.jgi.doe.gov/

2
https://www.ncbi.nlm.nih.gov/

## Supplementary Material

Supplementary Figure 1 *gSiBBM3* altered morphology. **(A)** Phenotypic growth differences between *gSiBBM3* lines without (left) and with (right) a full-length *gSiBBM3* transgene. **(B)** Ectopic expression of the *gSiBBM3* transgene in axillary bud (B) and young leaf (L) tissue.

Supplementary Table 1 Primers used for transgene construction, genotyping and transgene cloning and expression.

Supplementary Table 2 Cumulative data for the analysis of the three *gSiBBM* lines.
