# Supplementary figures and images for: Phylogenetically Distant BABY BOOM Genes From Setaria italica Induce Parthenogenesis in Rice
Source: Front Plant Sci. 2022 Jul 14;13:863908. doi: 10.3389/fpls.2022.863908 (PMC9329937; doi:10.3389/fpls.2022.863908)

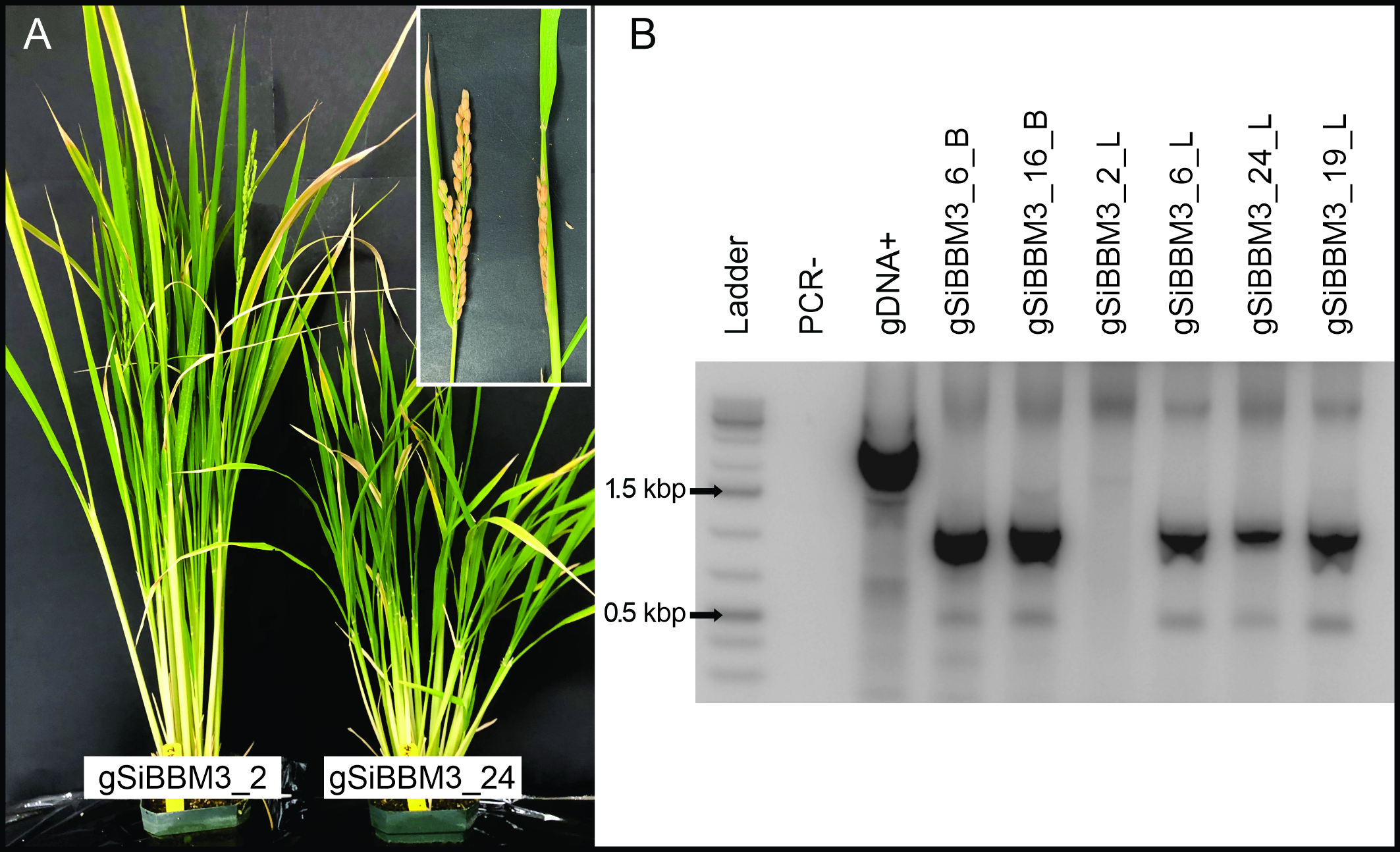

Supplement: Supplementary Figure 1 — gSiBBM3 altered morphology. (A) Phenotypic growth differences between gSiBBM3 lines without (left) and with (right) a full-length gSiBBM3 transgene. (B) Ectopic expression of the gSiBBM3 transgene in axillary bud (B) and young leaf (L) tissue. [file Image_1.JPEG]
