# Supplementary material for: Phylogenetically Distant BABY BOOM Genes From Setaria italica Induce Parthenogenesis in Rice
Source: Front Plant Sci. 2022 Jul 14;13:863908. doi: 10.3389/fpls.2022.863908 (PMC9329937; doi:10.3389/fpls.2022.863908)
Supplement: Supplementary Table 1 — Primers used for transgene construction, genotyping and transgene cloning and expression. [file Table_1.DOCX]

Supplemental Table 1: Primers used for transgene construction, genotyping and transgene cloning and expression.

| Primer name | Transgene | Purpose | Primer sequence | Tm (^o^C) |
| --- | --- | --- | --- | --- |
| SiB1-F | *gSiBBM1* | Construct | tacttaagATGGGTTCCACCAACAACTG | 65 |
| SiB1-R | *gSiBBM1* | Construct & Genotyping | ACGCTAAATCTGCACAACAAATGG | 61 |
| SiB2-5pF | *gSiBBM2* | Construct | tacttaagATGGCTACTGTGAACAACT | 62 |
| SiB2-5pR | *gSiBBM2* | Construct | AAATTTGTGGTCGTCGTGGGA | 61 |
| SiB2-3pF | *gSiBBM2* | Construct | CTGTTGTGCTTGATCTTTCCCCCAA | 65 |
| SiB2-3pR | *gSiBBM2* | Construct | GAGGATCCGGAAGCTCATTTTGGTGC | 65 |
| SiB3-5pF | *gSiBBM3* | Construct | tacttaagATGGCTTCCGCCAACAACTGGT | 65 |
| SiB3-5pR | *gSiBBM3* | Construct | ATTAGGGGCATAGTTAATGCGGC | 63 |
| SiB3-3pF | *gSiBBM3* | Construct | ATTCTTAAGACTAGCTAATGACTACG | 60 |
| SiB3-3pR | *gSiBBM3* | Construct | ATGTCGACTTCCCCTCACGGCAATGTTTTA | 65 |
| S81-F | *gSiBBM1 & gSiBBM3* | Genotyping | ATCCTTTCCCATTCCTCCCACTAA | 60 |
| S51-F | *gSiBBM2* | Genotyping | GTCTGCTTCAACATCCCCCA | 60 |
| S52-R | *gSiBBM2* | Genotyping | GCTATGCTCGAGTGAGGCAT | 60 |
| S11-R | *gSiBBM3* | Genotyping | ATTAGGGGCATAGTTAATGCGGC | 60 |
| Hygro - F | All | Genotyping | CGATCGACAGATCCGGTCGGC | 65 |
| Hygro - R | All | Genotyping | GGAGGCATCCGGAGCTTGCAG | 65 |
| ADF3-F |  | RT-PCR | TACAGTCCAAAAGGATGCACCG | 60 |
| ADF3-R |  | RT-PCR | GATCTTTGAGCGCATCGAGACT | 60 |
| SR81-F | *gSiBBM1* | RT-PCR | ATGGGTTCCACCAACAACTGG | 60 |
| SR81-R | *gSiBBM1* | RT-PCR | CCTATTCTTGCTTGCCACCTTC | 60 |
| SR51 -F | *gSiBBM2* | RT-PCR | GCGACGTCTGCTTCAACATC | 60 |
| SR51 -R | *gSiBBM2* | RT-PCR | CCTGCTCGTGACTCACCATT | 60 |
| SR11 -F | *gSiBBM3* | RT-PCR | CTGTCGATGAGCACGGGG | 60 |
| SR11 -R | *gSiBBM3* | RT-PCR | GTTGTAGACCGTCGAGCTCG | 60 |
| SS81 -F | *gSiBBM1* | Full length cds | ATGGGTTCCACCAACAACTG | 60 |
| SS81 -R | *gSiBBM1* | Full length cds | AGTGACAGAAGCAGCAAAACTCT | 60 |
| SS51 -F | *gSiBBM2* | Full length cds | ACAAAACAAAAACCCCAAAAAGAA | 60 |
| SS51 -R | *gSiBBM2* | Full length cds | TGCCATTAGTCTTCACCTCATGT | 60 |
| SS11 -F | *gSiBBM3* | Full length cds | AGAATACTTAAGATGGCTTCCGC | 60 |
| SS11a-R | *gSiBBM3* | Full length cds | GTGCATGCACGGGTTAATTGT | 60 |

Double underlined sequences denote the *AflII* site created for each construct while the yellow highlighted ATG is the first codon. Single underlined and dashed underlined sequence denotes a *BamHI* and *SalI* site created for cloning
